# Effects of a Modern Kefir on Conditions Associated with Moderate Severe Spastic Quadriparesis Cerebral Palsy

**DOI:** 10.3390/microorganisms10071291

**Published:** 2022-06-25

**Authors:** Adán Israel Rodríguez-Hernández, Eva Salinas, Deli Nazmín Tirado González, Carlos Velasco Benitez, Mariela Jiménez, Laura E Córdova-Dávalos, Daniel Cervantes-García, Victor Federico Rodríguez Nava, Luis G. Bermúdez-Humarán

**Affiliations:** 1 Departamento de Nutrición, Universidad Autónoma de Aguascalientes, Av, Universiades 940, Aguascalientes C.P. 20100, Mexico; israel.rodriguez@edu.uaa.mx; 2 Laboratorio de Inmunología, Departamento de Microbiología, Universidad Autónoma de Aguascalientes, Av, Universidad 940, Aguascalientes C.P. 20100, Mexico; emsalin@correo.uaa.mx (E.S.); mayojv@hotmail.com (M.J.); laura.cordova@edu.uaa.mx (L.E.C.-D.); dcervantesga@conacyt.mx (D.C.-G.); 3 Departamento de Ingenierías, Tecnológico Nacional de México (TecNM), Instituto Tecnológico El Llano Aguascalientes, Carr. Aguascalientes-S.L.P km 18.5, El Llano, Aguascalientes C.P. 20330, Mexico; deli.tg@llano.tecnm.mx; 4 Departamento de Pediatría Cali-Colombia, Universidad del Valle, Cali C.P. 76001, Colombia; carlos.velasco@correounivalle.edu.co; 5 Consejo Nacional de Ciencia y Tecnologia, Av. Insurgentes Sur 1582, Ciudad de México C.P. 03940, Mexico; 6 Departamento de Enfermería, Universidad Autónoma de Aguascalientes, Av, Universidad 940, Aguascalientes C.P. 20100, Mexico; victorrguez7@hotmail.com; 7 Micalis Institute, Université Paris-Saclay, INRAE, AgroParisTech, 78350 Jouy-en-Josas, France

**Keywords:** probiotics, cerebral palsy, digestive disorders, constipation, dyspepsia, common cold, malnutrition

## Abstract

Cerebral palsy (CP) in children constitutes a set of movement and body posture disorders caused by brain injury, which in turn is associated with a series of intestinal, respiratory, and malnutrition conditions. Twenty-four children were selected and included for the present study and subdivided into two groups: (1) children who included modern kefir (containing 12 probiotic species) in their diet; and (2) control group (not including kefir in their diet). The group supplemented with modern kefir received a beverage with multi probiotic species and the control group received commercial yogurt (which included the 2 typical lactic acid bacteria) for 7 weeks. Anthropometric variables, resting energy expenditure, presence, and diagnosis of functional digestive disorders (FDD), frequency of respiratory problems, presence of elevated C-reactive protein, differential count of leukocytes were evaluated. A significant increase in weight and height was found in the kefir group at the final time point. In addition, kefir intake promoted a significant reduction in functional constipation and stool hardness and increased the absolute value of blood lymphocytes. Since the fermented milk beverage modern kefir improves constipation, which is the most important FDD in children with CP and the nutritional and immune status, it could be considered an important strategy to improve health in these children.

## 1. Introduction

Cerebral palsy (CP) is a posture and movement disorder that results from damage to the immature brain or abnormal brain development. According to a recent report from the Center for Disease Control and Prevention (CDC) [1], it is estimated that the worldwide prevalence of CP ranges from 1 to almost 4 per 1000 newborns. In Mexico, there are around 500,000 people with CP and around 12,000 new cases are reported each year [2]. In the Rehabilitation Center Telethon (CRIT) in Aguascalientes, the registered population oscillates above 2000 patients in recent years, of which 110 have the problem of hypertonia or severe spasticity [3].

The CP is associated with various conditions such as malnutrition [4,5], alterations in swallowing mechanics [6], respiratory diseases [7], as well as inflammatory bowel diseases (IBD) [8,9] and constipation [9,10]. These conditions, in turn, determine a loss or little gain in body weight and a limited muscle mass gain, represented as poor growth and muscle atrophy [11]. Additionally, children with CP present neuromotor and gastrointestinal limitations that influence improper diet, such as poor mouth opening, respiratory secretions, poor chewing, etc. [12].

Different symptomatic manifestations of gastrointestinal disorders are present in children with CP. Besides, laboratory studies have confirmed that those children show different grades of inflammation, characterized by leukocytosis (an excess of leukocytes in the blood), elevated erythrocyte sedimentation rate, high blood levels of C-reactive protein (CRP) (a marker of an acute phase of inflammation), hypoalbuminemia, and anemia (which could be normocytic due to chronic disease, microcytic due to iron deficiency, or more frequently combined) [13,14].

In a study by Colson et al. [9] an incidence of 198 IBD cases per 100,000 children with CP was observed, with ulcerative colitis, Crohn’s disease, and indeterminate colitis the most frequently encountered types of IBD. Some of the symptoms were bloody stools and poor body weight gain. Recently, Roma Foundation published, through collaborators in Latin America, an update of the Rome IV Criteria for Functional Digestive Disorders (FDD) questionnaire in its Spanish version of three different pediatric age groups [15]. Through this tool, it was detected the presence of functional dyspepsia, epigastric pain syndrome, irritable bowel syndrome (IBS), functional abdominal pain, functional abdominal migraine, functional constipation, fecal incontinence, adolescent rumination syndrome, and aerophagia. It may be present one or several FDDs.

Malnutrition is common in CP and its prevalence is more common in spastic CP [4]. This condition is strongly related to a deficient response of the immune system [16], as an insufficient and poor-quality food intake translates into a deficit of nutrients for the synthesis of molecules or cells of the immune system. In addition, a correlation between a poor immune response, dysbiosis, and chronicity of respiratory infections has also been established [17].

Probiotics are “*live microorganisms that, when administered in adequate amounts, confer health benefits to the host*” [18]. Probiotic-based treatments to improve human health are associated with the “brain-gut-immune axis”, as an adequate microbiota benefits these three main systems [19]. Clinical trials have proven that the intake of probiotics reduces inflammation of the intestinal and respiratory mucosa, in addition to inducing other immunomodulatory properties [20,21,22]. To date, there have been few clinical trials that show an increase or improvement in weight or body mass index in children with CP that have been treated with probiotics, particularly children with a history of prematurity and poor neurological development [23,24,25].

Traditional kefir, also called milk kefir, is based on the fermentation of milk by kefir grains and includes several microorganisms. It is basically composed of a large variety of probiotic microorganisms, mainly bacteria from genera *Bifidobacterium*, *Lactococcus*, *Leuconostoc*, and *Lactobacillus*, as well as yeasts from *Saccharomyces*, containing an average range of up to 50 probiotic species [26,27,28]. Anti-inflammatory, immunomodulatory, antiviral, antimicrobial, and antifungal properties have been attributed to the intake of milk kefir [29,30,31]. A version of lyophilized or powdered kefir probiotic species can be added to conventional milk for the corresponding fermentation process, which is known as modern kefir. It contains an average of 10 to 14 probiotic species, mainly species of *Bifidobacterium*, *Lactobacillus*, and *Streptococcus*, but without yeasts [32,33,34]. In this sense, modern kefir is a modified version that has been designed to improve the organoleptic characteristics of traditional kefir while preserving most of the beneficial properties for host health [33,35]. Both traditional and modern kefir are similar in aspect to yogurt, but with better sensorial properties; nevertheless, only modern kefir guarantees the presence of 1 × 10^8^ colony-forming unit (CFU) of each probiotic species during the fermentation process [33].

The modern kefir of the Sello Rojo brand (Mexico), is a fermented dairy drink from the inoculation of 14 probiotic species isolated from traditional kefir [36]. Based on this argument, the objective of this study was to evaluate the effects of this modern beverage on the main pathological conditions associated with CP.

## 2. Materials and Methods

### 2.1. Subjects and Type of Study 

**Figure A1:**
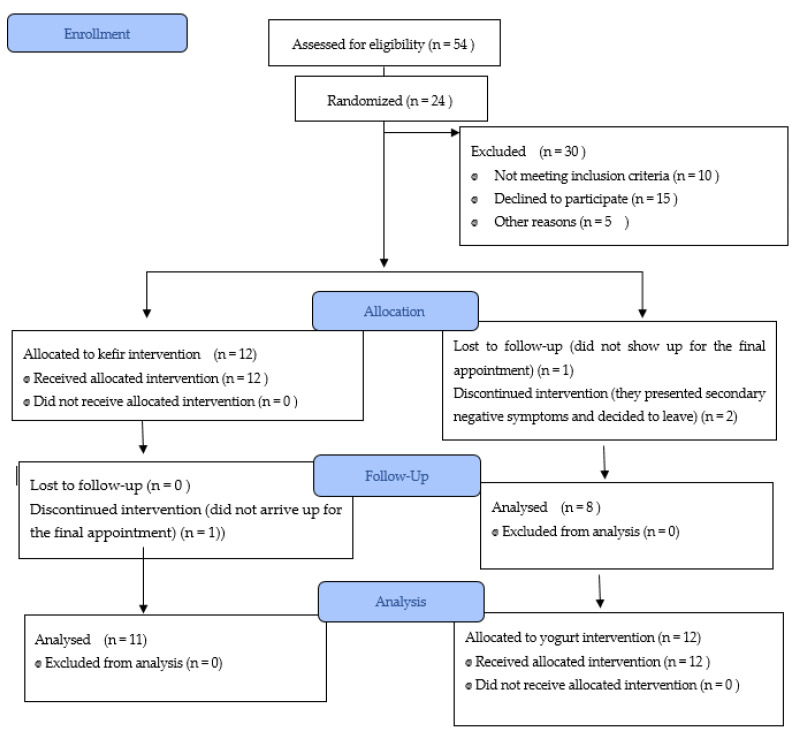
CONSORT 2010 Flow Diagram.

A double-blind randomized clinical trial was conducted on 24 children between 4 and 8 years of age with moderate severe spastic CP from the Rehabilitation Center Telethon (CRIT) in Aguascalientes, Mexico, who attended in the 2020–2021 period (Figure A1). Children included presented CP originated due to postnatal causes. Besides, they were diagnosed with III and IV Asworth spastic level, and IV Gross Motor Function Classification System (GMFCS). Those children who had basic pharmacological treatment and did not have a special diet (*ad libitum*) due to some comorbidities were included; however, those whose parents did not agree to participate or were on an antibiotic treatment were eliminated. Those subjects whose parents decided to withdraw from the study or did not meet the scheduled evaluation dates were eliminated. The sample size was calculated using the Lachenbruch formula for the calculation of the sample of “Difference of two proportions” with a confidence level of 90% and a power of 80%, with a 30% adjustment for losses using the free Win Epi calculator. A simple random probabilistic sampling (randomized) was applied, using the STATS 2.0 software. The “Random Number Generator” subprogram was used to automatically generate patient numbers. Participants were randomly assigned to control *(n* = 12) or model kefir (*n* = 12) groups. 

### 2.2. Treatment Composition and Clinical Trial Design

Commercial yogurt (kindly donated by Láctica Creativa, Mexico) was administered to the control group (yogurt group) and modern kefir (kindly gifted by Alimentos Sello Rojo S.A. of C.V, Mexico) to the experimental group (kefir group) for 49 days (7 weeks). The different evaluations were carried out at time zero and at week (W) number 7 of the treatment. Nutritional composition of the commercial yogurt was: 5.9 g of protein, 0.8 g of fat, 210 mg of calcium, 10.8 g of carbohydrates and 89 mg of sodium per 240 mL (100 calories). It also contained two probiotic species: *Streptococcus thermophilus*, and *Lactobacillus bulgaricus*. In addition, acesulfame-K (5 mg/100 mL) was included as sweetener. Nutritional composition of modern kefir was: 5.9 g of protein, 0.9 g of fat, 220 mg of calcium, 10.8 g of carbohydrates, 104 mg of sodium and 0.6 g of lactase per 240 mL (108.3 calories). The 12 probiotic species contained in modern kefirwere: (1) *Bifidobacterium infantis*, (2) *Bifidobacterium lactis*, (3) *Lactobacillus acidophilus*, (4) *Lactobacillus delbrueckii* spp. *lactis*, (5) *Lactobacillus fermentum*, (6) *Lactobacillus paracasei*, (7) *Lactobacillus rhamnosus*, (8) *Lactococcus lactis* spp. *cremoris*, (9) *Lactococcus lactis* spp. *lactis*, (10) *Lactococcus lactis* spp. *lactis biovar. diacetylactis*, (11) *Leuconostoc mesenteroides*, (12) *Leuconostoc pseudomesenteroides*. Sucralose (7.2 mg/100 g) was included as a sweetener. Participant of both groups were administered orally 250 mL of the corresponding beverage per day.

At the end of the study, a survey on the perception of effects and satisfaction of the product was applied to parents or guardians of children and was validated through a concordance test (test retest). A Likert scale survey was used to obtain responses such as: 1, particularly good; 2, good; 3, regular; 4, bad; 5, particularly bad.

### 2.3. Anthropometric Measurements

Height was obtained under the Stevenson et al. [37] segmental measurement criteria through the upper arm length equation while weight by using a wheelchair weighing scale (PWC-620 model, Tanita, Tokyo, Japan). In addition, body mass index (BMI) was calculated.

### 2.4. Evaluation of Resting Energy Expenditure

Resting energy expenditure (REE) was measured through the indirect calorimetry technique with a CardioCoach calorimeter (KORR Medical Technology, Salt Lake City, UT, USA), using the petit mask for children. Subjects were evaluated for 15 min in a reclining chair.

### 2.5. Diagnosis of Intestinal Disorders and Respiratory Problems in Children 

**Figure A2:**
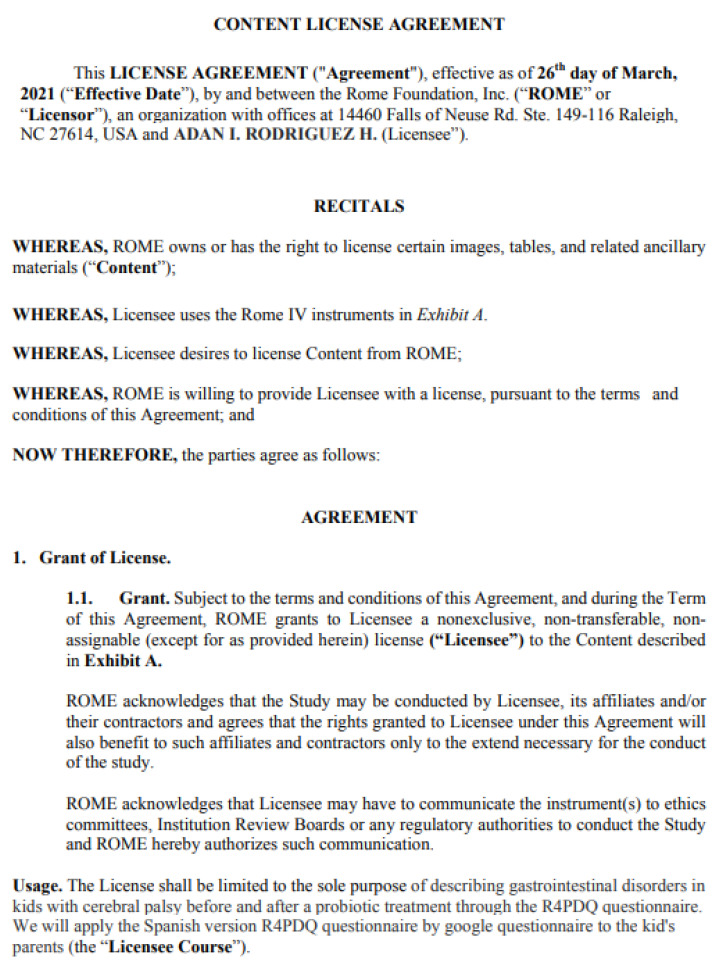
The Rome Criteria (IV) of functional digestive disorders in clinical practice, Content License Agreement.

A digital survey was applied before (W0) and after (W7) treatment administration using the FDD questionnaire under the Rome IV Criteria (Figure A2) for parents or guardians of children between 4 and 10 years of age in the Latin American Spanish language and under the legal permission of the foundation. This instrument could determine the presence or absence of up to 12 disorders. Besides, this survey included an item about stool consistence and parent responses were evaluated using Bristol scale, in which feces are classified in 7 types as follows: type 1, separate hard lumps like nuts; type 2, sausage-shape but lumpy; type 3, like a sausage but with cracks on its surface; type 4, like a sausage or snake, smooth and soft; type 5, soft blobs with clear-cut edges; type 6, mushy consistency with ragged edges; type 7, liquid consistency with no solid pieces; type 8, it is always different.

Other digital survey was applied under the criteria of the program “Integrated attention to prevalent childhood diseases” to know the frequency of respiratory problems for periods ranging from weekly to monthly.

The perception of the beneficial digestive and respiratory effects after applying the treatments was evaluated through a digital survey applied to parents or guardians of children, in which a Liker scale mentioned 5 items: 1, particularly good; 2, good; 3, regular; 4, bad; 5, particularly bad.

### 2.6. Blood Leukocyte Count and Serum CRP Analysis

For differential (lymphocytes, monocytes, and granulocytes) count of leucocytes, five mL of blood was drawn by venipuncture and collected in tubes with EDTA (Vacutest Kima, Italy). Two hundred μL of each blood sample were evaluated in an automated hematologic analyzer Orphée Mythic 18 (Diamond Diagnostics, Holliston, MA, USA). Clinical abnormalities in white cells, such as leukocytosis, lymphocytosis, leukopenia, or lymphocytopenia, were diagnosed using the 2018 Spanish pediatric hematology guidelines [38].

To the qualitative (negative or positive) determination of CRP, 1 mL of undiluted serum was analyzed by latex agglutination plate test (LICON, Mexico). A negative result indicates that the CRP serum level was lower than 6 mg/L (healthy condition) and a positive result that it was higher than 6 mg/L (inflammatory condition).

### 2.7. Statistical Analysis

Statistical analysis was performed using the SPSS software (SPSS Inc., Chicago, IL, USA). Comparisons among groups were made with the chi-square test to compare non-parametric type variables with respect to baseline and final time between independent samples, McNemar test for analyzing dichotomous variables, the Wilcoxon test for ordinal variables, and Mann Whitney U test for related samples. Meanwhile, to analyze parametric variables a Student’s *t*-test was used for independent samples and related samples. For the correlation of the variables of presence or absence of FDD and the perception of beneficial effects of the parents after treatment, a Pearson’s R was used, where R = 0.4–0.9. Differences were considered statistically significant when *p <* 0.05. 

## 3. Results

### 3.1. Characteristics of the Subjects

From the 24 patients that were included in this study, just 19 completed the treatments. The average age was 6.05 years, and 36.8% were female and 63.2% male. The level of classification of gross motor function was group 4 in 100% of the population, while the distribution based on the degree of hypertonia according to the Asworth scale was: 21.1% were classified as grade 3 (moderate), 36.8% as grade 4 (severe) and 68.4% as grade 3–4 (moderate severe). No significant differences were found between the variables age, weight, height, BMI, and REE at the beginning of the study, as shown in Table 1.

Regarding the anthropometric parameters and the needs of energy expenditure at rest or REE, no statistically responses were found using the Student’s *t*-test among the independent samples of the groups “before” and “after” the study. However, when the Student’s *t*-test from related samples (that compared data before (W0) and after (W7) the treatment) showed that not only the height of kefir group increased significantly (*p* = 0.003) but also the weight (*p* = 0.027) as is shown in Table 2. In addition, we found a significantly positive correlation between Weight W0 and W7 with REE W0 and W7 (r = 789, *p* = 0.003, Spearman r).

Regarding the results for the weight, we also found a significant negative correlation among the diagnostic Asworth hypertonic grade and the weight and BMI, which means that the higher the hypertonia is related to a lower body weight (r = 0.6) or BMI (r = 0.8) (*p* < 0.01, Pearson r).

### 3.2. Effects of Kefir Administration on Functional Digestive Disorders

**Figure 1:**
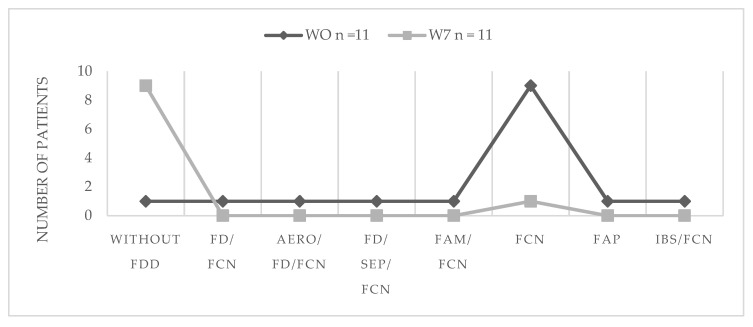
Presence of functional digestive disorders before and after treatment with modern kefir. FDD, functional digestive disorders; FCN, functional constipation; FD, functional dyspepsia, FN functional nausea FN; FAM, functional abdominal migraine; FAP, functional abdominal pain; IBS, irritable bowel syndrome; SEP, syndrome epigastrium pain.

Figure 1 shows the prevalence of different FDDs in the kefir group before and after the intervention. The most recurrent FDD detected at W0 was the functional constipation with a prevalence of 90.9% (*n* = 10) from those 45.45% presented as a unique disorder (not accompanied by another FDD), followed by functional dyspepsia (27.3%; *n* = 3), which in most cases was accompanied by other FDDs, such as aerophagia (9.1%, *n* = 1), and pain syndrome in epigastrium (9.1%, *n* = 1) combined with functional constipation. One of the children presented functional abdominal migraine and other IBS, accompanied by constipation. Finally, functional abdominal pain was diagnosed in other participant as a sole disorder. Interestingly, there was a decrease in the presence of FDDs detected after the intervention with modern kefir. In addition, it was observed a notable reduction in constipation, going from 90.9% to just 9.1%, and those children that had some other FDD, no longer was present after 7 weeks of kefir intake.

**Figure 2:**
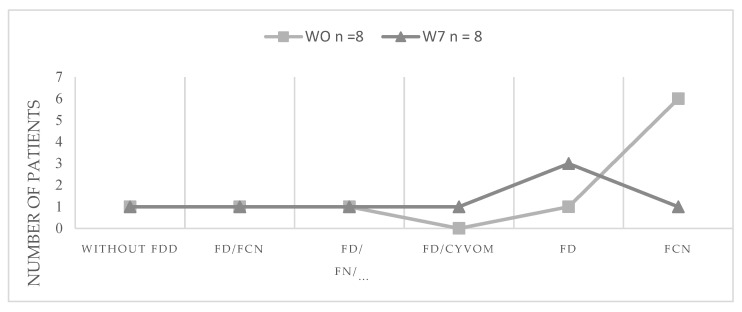
Presence of functional digestive disorders before and after treatment with conventional yogurt. FDD, functional digestive disorders; FCN, functional constipation; FD, functional dyspepsia; FN, functional nausea; CYVON, cyclic vomiting.

On the other hand, 75% of the control group (*n* = 6) presented constipation at the beginning of the study but the proportion was reduced to 12.5% (*n* = 1) after the treatment with kefir (Figure 2). However, from the 37.5% of the cases that presented functional dyspepsia, 12.5% who presented functional nausea at the beginning of the study did not improve after the kefir intake. In addition, an unexpected finding was the appearance of a case of dyspepsia and cyclic vomiting syndrome in one of the patients.

Table 3 shows that the most reported cases of FDD in kefir group at week 0 or basal time were not present at the end of the study in 100% of subjects, with the exception of functional constipation that shows the 88.8% of the recovery. Only in the case of functional constipation, the decreasing of the cases mediated by kefir intake was statistically significant due to the high number of cases present at baseline time.

**Figure 3:**
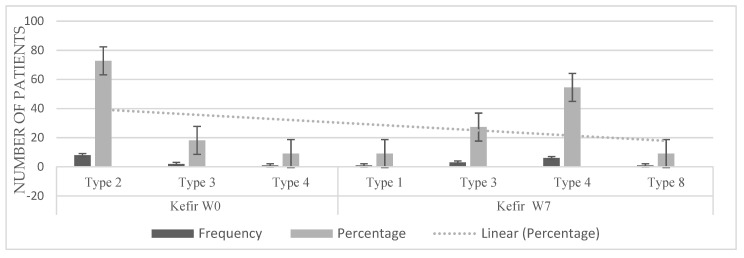
Differences in stool consistency according to the Bristol scale before and after consumption of modern kefir. Wilcoxon test *p* = 0.007.

**Figure 4:**
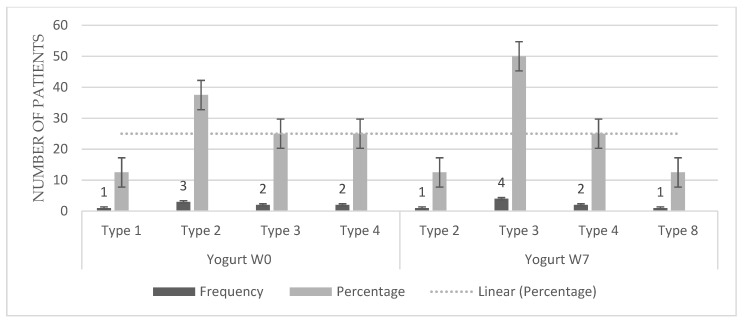
Differences in stool consistency according to the Bristol scale before and after consumption of commercial yogurt. Wilcoxon Test *p* = 0.167.

Regarding the evaluation of stool consistency using Bristol scale, a statistically significant improvement was found in kefir group using the Wilcoxon test for ordinal variables (*p* = 0.007). Specifically, there was a reduction in severity of the hardness of the stool (Figure 3), as the majority of the population (72.7%) had stools type 2, sausage-shape but lumpy (almost the worst level of hardness) before the intake of kefir, but at the end of the treatment the majority of children presented smooth and soft elongated stools (54.5%) or other lower levels of stool hardness. Only in one subject there was no improvement (9.1%). On the contrary, in the yogurt group (Figure 4) there were not significant improvement in stool consistency.

On the other hand, when it was compared the perception of the included children’s parents at the end of the study, regarding the gastrointestinal benefits of the kefir addition in diets, 63.6% of them perceived the kefir treatment as particularly good but no one of the in the yogurt group perceived the treatment as particularly good, the 25% of kefir group qualified it as good and 37.5% as regular. However, the difference between groups using the Mann Whitney U test at the end of the study was not significant (*p* = 0.103).

### 3.3. Effects of Kefir Administration on Respiratory Problems

Table 4 shows that there were significant differences by chi^2^ test between W0 and W7 in relation to the presence of the frequency of respiratory problems that ranged from higher to lower frequency on an ordinal scale per week, per month, at two or 3 months, twice a year and never. However, these results were not significant by Wilcoxon test. 

**Figure 5:**
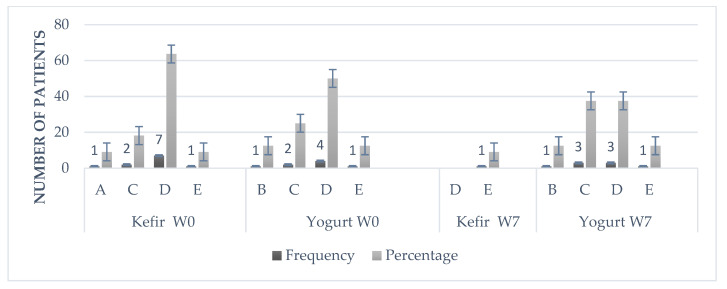
Frequency of the common cold at week 0 and week 7 of the intervention. A, weekly; B, monthly; C, every 2 months; D, 2 times per year; E, never.

Figure 5 shows that in the kefir group, the small population that got sick every week or every 2 months before treatment (W0), reduced the presence of colds in 100% of the cases compared to the end of the kefir treatment (W7). There was no change in cold frequency in the control group.

On the other hand, when comparing the effect that the treatment had according to the perception of improvement in the presence of respiratory problems between the two treated groups, the difference was not significant (*p* = 0.06). However, 45.5% of the parents of the patients treated in the kefir group referred to the product as particularly good vs. 12.5% of the control group.

### 3.4. Effect of kefir Administration on the Differential Count of Leukocytes and Serum CRP Level

**Figure 6:**
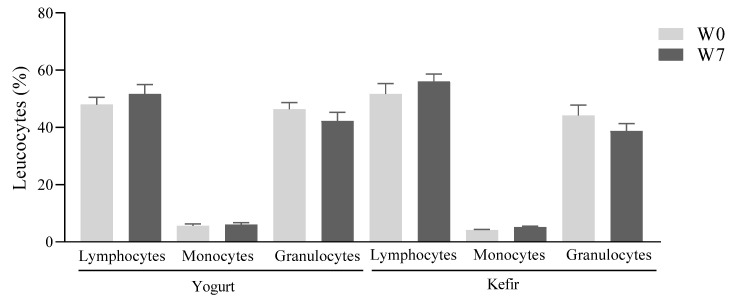
Effect of Kefir on relative differential values of leukocytes in peripheral blood. The percentages of lymphocytes, monocytes and granulocytes were analyzed. Data are expressed as mean ± standard error of mean; *n* = 7 yogurt group and *n* = 9 kefir group.

The percentage of lymphocytes, monocytes, and granulocytes of the patients in each group before and after treatment with yogurt or kefir remained unchanged (Figure 6). Likewise, there was no change in the percentage of each leucocyte at weeks 0 and 7 when comparing between groups.

**Figure 7:**
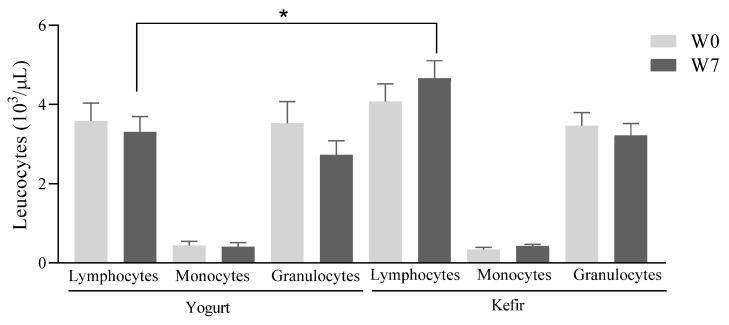
Effect of Kefir on absolute differential values of leukocytes in peripheral blood. The absolute number of lymphocytes, monocytes and granulocytes were analyzed. Data are expressed as mean ± standard error of mean; *n* = 7 yogurt group and *n* = 9 kefir group, * *p* < 0.05.

On the other hand, there were no significant differences in the absolute values of monocytes, granulocytes, and lymphocytes of the patients in each group before and after treatment with yogurt or kefir (Figure 7). Interestingly, the absolute value of lymphocytes in children that received treatment with kefir for 7 weeks (W7) was 40.82% higher than in those treated with yogurt for the same time (4.66 ± 0.43 vs. 3.31 ± 0.38 × 10^3^ lymphocytes/µL of blood, *p* < 0.05), with no differences between the absolute values of these cells in the patients of both groups before starting treatment (W0; 4.07 ± 0.44 vs. 3.58 ± 0.45 × 10^3^ lymphocytes/µL of blood, respectively). The absolute number of monocytes and granulocytes was similar when comparing the values obtained before (W0) and after (W7) the treatments between the groups.

Regarding the total count of lymphocytes in kefir group, although there was not significant change between W0 and W7, it is worthy to mention the case of a child that presented less than 2000 lymphocytes/μL (which indicates the risk of immunodepleting or mild protein malnutrition [39]) at W0, but increased considerably at W7.

In relation to serum CPR analysis, it was found a positive result (CRP > 0.06 mg/dL according to the LICON qualitative Test) in one child of the kefir population at W1 and W7, while all children in the yogurt group were negative. It has been reported CRP values above 17.1 mg/dL are associated with IBD [40]. However, as the test we used is a qualitative type, we cannot confirm the association among these two criteria. In this regard, we found significant correlation between the presence of IBS and CRP in W0 (r = 1, *p* < 0.005; Spearman r)

### 3.5. Unexpected Findings and Secondary Effects

Unexpected side effects were found in the commercial yogurt group, an exacerbation of gastroesophageal reflux was observed in a controlled patient who presented cyclic vomiting syndrome at the end of the study, as well as the appearance of two cases of dyspepsia. In the kefir group, one case of exacerbation of constipation was reported at the end of the study. No subjects excluded due to severity or respiratory complications were reported during the study.

Regarding the hematological markers not included in present study, after the blood analysis it was found the presence of hypochromic anemia before and after treatments in both kefir (54.7%) and yogurt (87.5%) groups, as well as granulocytopenia at 36% in kefir and 25% in yogurt group.

## 4. Discussion

Our results are similar to those reported in previous studies that have described poor weight gain and growth in children with CP [11,16,41,42,43]. Therefore, the weight gain obtained in the kefir group represents an important strategy to contribute to nutritional status in this population of children. According to Krick et al. [41], the weight-for-age curves in children with spastic quadriparesis show an annual weight gain trend of 1 kg per year (approximately) between 4 and 10 years of age. This means that the average gain of 375 g in the kefir group during the 7 weeks of exposure to treatment represents a considerable benefit. It has also been reported an increase in the weight of infants diagnosed with the highest risk of neurological damage and treated with various probiotic species such as *Bifidobacterium infantis*, *Streptococcus thermophilus*, *and Bifidobacterium lactis* [24,25]. Garcia et al. [44] found no significant differences in the weight of children with CP treated with pre-, pro-and synbiotics (*Lactobacillus reuteri DSM 17938*, and Agave inulin as a prebiotic). Moreover, it has been described that weight is lower in ambulatory children with a higher degree of athetosis, which is caused to energy expenditure due to this alteration in muscle tone [45]. However, this study also revealed a significant correlation between having a higher degree of hypertonia or spasticity and having a lower weight or body mass index. Results on the weight status of children with CP are sometimes controversial, while Perenc et al. [4] reported an association between greater motor disability and low weight and nutritional risk Martínez de Zabarte et al. [5] showed that the weight of children with CP tends to be in excess, although no significant differences were reported between overweight and GMFCS grade IV.

Regarding the REE, a relationship between the weight gain and the increase in REE has been reported in children with spastic quadriparesis CP in a malnutrition state who underwent in-hospital nutritional treatment [46], while a decrease in hypertonia but not a reduction in REE was shown in patients underwent physical and robotic therapy treatment [47]. Our results agree with those reported by García et al. [46], since in the present study an association was found between weight gain and height, and with the increase in REE, when the children were treated with kefir. An inverse correlation was also found between the hypertonic degree and BMI, so that the greater the hypertonia the lower the BMI.

About the effect of kefir on FDDs, there is no similar type of study in children with disabilities, due to the fact that the diagnostic criteria and the version of the measurement instrument in Latin American Spanish of the Roma Foundation have been recently updated. However, different studies have suggested or reported the efficacy of the use of probiotics in the treatment of FDD in children, such is the case of IBS, functional dyspepsia, abdominal pain and migraine, mostly in controlled clinical trials using a single strain or combination with prebiotics [48,49,50,51]. Although studies with multi-probiotic species intervention in children are limited, Guandalini et al. [50] reported the benefits of various combinations of probiotic bacteria, including *Bifidobacterium breve*, *Bifidobacterium longum*, *Bifidobacterium infantis*, *Lactobacillus acidophilus*, *Lactobacillus plantarum*, *Lactobacillus casei*, *Lactobacillus bulgaris*, *and Streptococcus thermophilus* (a formulation currently available as Vivomixx in Europe and Visbiome in the US and very similar to the modern kefir formulation), on dyspepsia, IBS, functional abdominal pain, flatulence, bloating, among others. Interestingly, our results show that the few cases of dyspepsia, aerophagia, irritable bowel, functional abdominal pain and migraine, as well as several of these FDDs grouped into functional constipation that were diagnosed in children with CP at the beginning of the study, did not appear at the end of the modern kefir treatment, which corroborates that the supplementation with various combinations of probiotic bacteria contributes to the reduction in symptoms of various FDDs. In this same sense, it has also been described that traditional kefir helps to eradicate conditions such as dyspepsia and irritable bowel [52]. On the other hand, in a study carried out by Colson et al. [9], it was reported an incidence of 198 cases of IBD per 100,000 children with CP, that is to say a higher prevalence than in the generall population. The unique case of IBD diagnosed in one child with CP in our study, showed them getting significantly better after modern kefir treatment. Strikingly, it was the only children with a positive CRP test, corroborating the association between IBD and CRP previously described [40]. Although IBD was not present after modern kefir treatment, CRP test was still positive. However, it is important to consider that we used a qualitative test, so we can not exclude that modern kefir treatment decreased CRP levels, as it has been described to traditional kefir [53].

Particularly, in children with CP and chronic constipation, it has been reported that different probiotics contribute significantly to the reduction in this FDD, mainly with the strain *L. reuteri* DSM 17938 (1 × 10^8^ UFC) combined with inulin as a prebiotic [44]. However, multi probiotic species combinations studies are limited. Our results show that constipation and stool consistency were significantly improved in children with CP treated with modern kefir. In adults, there is also evidence of the efficacy of multi-probiotics species treatment and a prebiotic (fructooligosaccharide) against constipation [54], using a granulated multi-probiotic species preparation (Hexbio) containing *Lactobacillus acidophilus*, *Lactobacillus casei*, *Lactobacillus lactis*, *Bifidobacterium bifidum*, *Bifidobacterium longum*, *and Bifidobacterium infantis*, (with 3 × 10^10^ cfu per envelope) [54], or through the formulation of the strains *L. paracasei Lpc-37*, *B. lactis Bl-04*, *L. acidophilus La-14*, *and L. plantarum Lp-115* [55]. On the other hand, reflux (that under the Rome IV criteria is called functional vomiting syndrome) usually occurs in children with CP [8,10,56]. However, in the present study, it occurred in a reduced number of participants and in two cases of the control group that consumed conventional yogurt for 7 weeks, an unexpected finding that was considered as a negative effect of commercial yogurt in this population.

Regarding respiratory infections, the use of traditional kefir has shown significant effects in reducing the frequency of respiratory problems and complications, even against SARS-CoV2 [57]. In our study, treatment exposure and the size of the sample were not enough to show an association between modern kefir intake and the reduction in respiratory infections. Moreover, it is known that the diversity of species contained in traditional kefir can reach more than 50 probiotics species [57]. Particularly, the reduction in the frequency of viral respiratory infections and of the duration of related symptoms, including the common cold, has been associated with treatments composed of species of the *lactobacillus genus*: *L. acidophilus*, *L. rhamnosus*, *L. fermentum*, *L. plantarum*, *Lactobacillus delbrueckii* spp. *Lactis*, as well as bifidobacteria, *Lactococcus lactis* spp. *cremoris*, and *Streptococcus thermophilus* [58]. Thus, the cases that showed reduction in common cold in the kefir group of the present study could be associated with the presence of similar probiotic species in the modern kefir, and although the results were not significant, 100% of the cases in which the common cold occurred frequently from one week to two months reduced this respiratory problem.

Different works have reported an association between kefir intake and lymphocyte number. Himada et al. [57] showed an increase and improvement in the production of lymphocytes after the consumption of traditional kefir, and Kranewitter et al. [59] reported a growth and increase in the number of lymphocytes, especially CD4 and CD8 lymphocytes. Besides, the treatment with a kefir designed from lyophilizates of traditional kefir bacteria showed an increase in the number of lymphocytes at the level of intestinal villi [60]. Our results are in accordance with the mentioned works, since it was possible to demonstrate an increase in the absolute number of lymphocytes in the kefir group. This effect of modern kefir might be mediating the decrease in the frequency of respiratory problems observed in children with CP, as it has been reported that a reduction in lymphocytes is associated with greater severity and poor prognosis of respiratory infections [61], even now used for prognosis of complications from COVID-19. It is know that intestinal microbiota can exert an important immunoregulatory activity on airway immune response through intestinal dendritic cell activation that can be translocated to lungs and induce the differentiation of regulatory T cells, and by producing short-chain fatty acids (SCFAs) that influence the epithelial and immune cells for a better adaptive immune response [22]. In this sense, traditional kefir intake by adult individuals has been associated with an increase in the levels of SCFAs in plasma and intestinal lumen, especially butyrate, and to a reduced amount of circulating lipopolysaccharide [62,63]. Probiotics have the ability to positively modulate the composition of the intestinal microbiota and hence reduce intestinal permeability, oxidative stress and inflammation [64,65,66,67]. Particularly, individual kefir probiotic species, such as *Bifidobacteria*, improving gut barrier function, and attenuate bacterial/endotoxin translocation [68]. Whether modern kefir induces a change on gut microbiome of children with CP needs to be investigated. Thus, the aim of our current research is to evaluate microbiome and quantify SCFAs from children fecal samples included in this study.

Finally, we found clinical evidence of hypochromic anemia and granulocytopenia in a high percentage of the participating children with CP. Iron deficiencies and anemia have been reported in children with CP [4,69,70], specifically relating this deficit to a greater degree of disability. The other unexpected finding was granulocytopenia, a condition not yet described for the population with CP. Although more research is needed, it could be associated with the intake of multiple drugs by children, as it usually occurs in this population due to various associated pathological conditions, including epilepsy [71].

## 5. Conclusions

In children with infantile CP, the addition in diets of a dairy-fermented drink called modern kefir, which contains 12 probiotics species might be a strategy not only for gaining body weight but also for reducing some digestive disorders, particularly the functional constipation, as well as inducing a positive regulation of the immune status that includes an increase in the total number of lymphocytes. These positive effects might contribute in the short or medium term to a better quality of life of children with CP. The administration of modern kefir might also be considered as a potential treatment in other conditions with immune or intestinal dysfunction.

## Figures and Tables

**Table 1:** Baseline characteristics of the study population.

	Yogurt Group *n* = 8	Kefir Group *n* = 11	p *
Age	5.88 (4–9)	6.18 (4–8)	0.907
Weight	14.21 (11.30–23.3)	14.41 (9.1–18.7)	0.590
Height	1.00 (0.90–0.122)	1.03 (0.91–1.22)	0.399
BMI	13.89 (11.74–15.65)	13.30 (11.15–15.32)	0.488
REE	882 (677–1093)	992.72 (732–1252)	0.176

* Student’s *t*-test. Data are expressed as mean and minimum and maximum value.

**Table 2:** Anthropometric parameters and rest energy expenditure (REE) of the study population at initial (W0) and final 7 (W7) time.

	Weight (g)				Height (cm)				BMI (kg/m^2^)				REE (Kcal)			
	W0	W7	*p* *	*p* **	W0	W7	*p* *	*p* **	W0	W7	*p* *	*p* **	W0	W7	*p* *	*p* **
Yogurt (*n* = 8)	14.21 ±1.45	14.40 ±1.57		0.19	1.00 ± 0.11	1.01 ± 0.04		0.06	13.89 ± 0.46	13.80 ± 0.47		0.47	882.02 ± 52.08	835.25 ± 71.42		0.26
Kefir (*n* = 11)	14.41 ± 0.91	14.77 ± 1.00		0.02	1.03 ± 0.02	1.045 ± 0.02		0.003	13.35 ± 0.40	13.36 ± 0.40		0.34	992.72 ± 58.72	1001.54 ± 74.61		0.84
			0.84				0.51				0.48				0.12	

W, week; Data are expressed as mean ± SEM. * Student’s *t*-test for independent samples ** Student’s *t*-test for related samples.

**Table 3:** Comparison of functional digestive disorders present at week 0 and week 7 of treatment.

Functional Digestive Disorders under Rome IV Criteria								
	YOGURT				KEFIR			
	Cases W0	Cases W7	% **	p *	Cases W0	Cases W7	% **	p *
Functional constipation	6	1	83.3	0.5	9	1	88.8	0.016
Functional dyspepsia/postprandial distress syndrome	3	4	1CI	1.00	3	0	100	1.00
Epigastric pain syndrome	0	0	-	*-*	1	0	100	*-*
Abdominal functional migraine	0	0	-	*-*	1	0	100	*-*
Functional abdominal pain	0	0	-	*-*	1	0	100	*-*
Irritable bowel syndrome	0	0	-	*-*	1	0	100	*-*
Functional nausea	1	1	0		0	0	-	*-*
Cyclic vomiting syndrome	0	1	1CI	*-*	0	0	-	*-*
Aerophagia	0	0	-	*-*	1	0	100	*-*

* McNemar test; ** FDD improvement percentage (W0 versus W7); 1CI, one case increment.

**Table 4:** Comparison of significant differences for the presence of respiratory problems according to the IMCI criteria.

		Frequency of Throat Infections W0 vs. W7	Frequency of Ear Infections W0 vs. W7	Frequency of the Common Cold W0 vs. W7	Frequency of Cough or Shortness of Breath W0 vs. W7	Frequency of the Presence of Respiratory Secretions W0 vs. W7
KEFIR	p *	0.081	0.003	0.012	0.39	0.285
	p **	0.157	0.317	0.257	0.157	1.000
YOGURT	p *	0.480	0.064	0.101	1.000	0.490
	p **	0.157	0.317	0.564	0.157	0.083

p * Chi^2^ test for differences between independent groups, p ** Wilcoxon signed rank test, asymptotic significance bilateral.
